# ANATOMIC VARIATIONS OF HEPATIC ARTERY: A STUDY IN 479 LIVER TRANSPLANTATIONS

**DOI:** 10.1590/0102-6720201700010010

**Published:** 2017

**Authors:** Olival Cirilo Lucena da FONSECA-NETO, Heloise Caroline de Souza LIMA, Priscylla RABELO, Paulo Sérgio Vieira de MELO, Américo Gusmão AMORIM, Cláudio Moura LACERDA

**Affiliations:** University Hospital Oswaldo Cruz, Faculty of Medical Sciences of Pernambuco University, Recife, PE, Brazil.

**Keywords:** Hepatic artery, Liver transplantation, Anatomic variations.

## Abstract

**Background:**

The incidence of anatomic variations of hepatic artery ranges from 20-50% in different series. Variations are especially important in the context of liver orthotopic transplantation, since, besides being an ideal opportunity for surgical anatomical study, their precise identification is crucial to the success of the procedure.

**Aim:**

To identify the anatomical variations in the hepatic arterial system in hepatic transplantation.

**Methods:**

479 medical records of transplanted adult patients in the 13-year period were retrospectively analyzed, and collected data on hepatic arterial anatomy of the deceased donor.

**Results:**

It was identified normal hepatic arterial anatomy in 416 donors (86.84%). The other 63 patients (13.15%) showed some variation. According to the Michels classification, the most frequently observed abnormalities were: right hepatic artery branch of superior mesenteric artery (Type III, n=27, 5.63%); left hepatic artery branch of the left gastric artery (Type II, n=13, 2.71%); right hepatic artery arising from the superior mesenteric artery associated with the left hepatic artery arising from the left gastric artery (Type IV, n=4, 0.83%). Similarly, in relation to Hiatt classification, the most prevalent changes were: right hepatic accessory artery or substitute of the superior mesenteric artery (Type III, n=28, 6.05%)), followed by liver ancillary left artery or replacement of gastric artery left (Type II, n=16, 3.34. Fourteen donors (2.92%) showed no anatomical abnormalities defined in classifications, the highest frequency being hepatomesenteric trunk identified in five (01.04%).

**Conclusion:**

Detailed knowledge of the variations of hepatic arterial anatomy is of utmost importance to surgeons who perform approaches in this area, particularly in liver transplantation, since their identification and proper management are critical to the success of the procedure.

## RESUMO

**Racional:**

A incidência das variações anatômicas da artéria hepática varia de 20-50% em diferentes casuísticas. Elas são especialmente importantes no contexto do transplante ortotópico hepático, visto que, além de representar oportunidade ideal para seu estudo anatômico cirúrgico, a sua precisa identificação é determinante para o sucesso do procedimento.

**Objetivo:**

Identificar as variações anatômicas no sistema arterial hepático em transplantes hepáticos.

**Método:**

Foram analisados retrospectivamente, no período de 13 anos, 479 prontuários de pacientes adultos transplantados, sendo coletados dados referentes à anatomia arterial hepática do doador falecido.

**Resultados:**

Identificou-se anatomia arterial hepática normal em 416 doadores (86,84%). Os outros 63 indivíduos (13,15%) apresentaram alguma variação. De acordo com a classificação de Michels, as anomalias mais frequentes foram: artéria hepática direita ramo da artéria mesentérica superior (Tipo III, n=27, 5,63%); artéria hepática esquerda ramo da artéria gástrica esquerda (Tipo II, n=13, 2,71%); artéria hepática direita ramo da artéria mesentérica superior associada à artéria hepática esquerda ramo da artéria gástrica esquerda (Tipo IV, n=4, 0,83%). Do mesmo modo, em relação à Classificação de Hiatt, as variações mais prevalentes foram: artéria hepática direita acessória ou substituta da artéria mesentérica superior (Tipo III, n=28, 6,05%), seguida da artéria hepática esquerda acessória ou substituta da artéria gástrica esquerda (Tipo II, n=16, 3,34%). Quatorze pessoas (2,92%) apresentaram alterações anatômicas sem classificação definida, sendo a de maior frequência o tronco hepatomesentérico, identificado em cinco (1,04%).

**Conclusão:**

O conhecimento detalhado das variações da anatomia arterial hepática é de grande importância aos cirurgiões que realizam abordagens nessa região, em especial no transplante hepático, visto que sua identificação e correto manejo são fundamentais para o êxito do procedimento.

## INTRODUCTION

Hepatic arterial anatomy is studied for many centuries, receiving attention from great scholars like Aristotle and Galen. However, only in the eighteenth century with Jacques Benigne Winslow and Albert Haller - considered the fathers of modern angiology - its blood irrigation was set correctly and from that, many anatomical anomalies were identified^3^ ^,^ ^4^ ^,^ ^6^.

These modifications are especially important in the context of orthotopic liver transplantation, since, besides being ideal opportunity for its surgical anatomical study, their precise identification is crucial to the success of procedure^1^ ^,^ ^3^ ^,^ ^4^ ^,^ ^14^. According to the literature, the incidence of anatomic variations of hepatic artery range from 20-50% in different series^1^ ^,^ ^4^.

The purpose of this study was to identify the anatomical variations in the hepatic arterial system in a cohort of 479 transplants.

## METHOD

Were retrospectively analyzed 517 transplant records of adult patients in a sample of 1063 transplants performed by the Liver Transplant Unit, University Hospital Oswaldo Cruz, Recife, PE, Brazil in a 13-year period - January 2002 to August 2015. Data for liver arterial anatomy deceased donor through the analysis of descriptions of the liver graft during transplantation, were collected. Of these, 38 were excluded by the absence of information in medical records.

Was adopted as anatomical vascular normality: emergence from common hepatic artery originating from the celiac trunk and - after the branch of the gastroduodenal artery - the proper hepatic artery, which divides into right and left hepatic artery in the hepatic hilum. When two hepatic arteries supply the same lobe, with one originating from the common hepatic trunk and the other from a separate arterial trunk, it was named as accessory artery.

Anatomical variations were classified according to Michels classification^8^ and its modification by Hiatt^8^ (Table 1).

**TABLE 1:** Michels^8^ and Hiatt^5^ classifications for variations found in hepatic arterial anatomy

Hepatic arterial anatomy	Michels classification	Hiatt classification
Normal anatomy	Type I	Type I
LHA branch LGA	Type II	Type II
RHA branch SMA	Type III	Type III
Type I and II association	Type IV	Type IV
LHA accessory LGA	Type V	Type II
RHA accessory SMA	Type VI	Type III
LHA accessory LGA + RHA accessory SMA	Type VII	Type IV
LHA accessory LGA+ RHA branch SMA	Type VIII	Type IV
CHA branch SMA	Type IX	Type V
RHA and LHA branch LGA	Type X	------
CHA aorta branch	--------	Type VI

"------"= liver variation not present in the corresponding classification column. RHA=right hepatic artery; LHA=left hepatic artery; SMA=superior mesenteric artery; LGA = left gastric artery; CHA=common hepatic artery

## RESULTS

Among 479 liver transplants were identified 416 donors in normal hepatic arterial anatomy, corresponding to 86.84% of the sample (Type I). The other 63 patients (13.15%) had some anatomical variation (Table 2).

**TABLE 2:** Changes found in hepatic arterial anatomy

Anatomical variations	n	%	Michels classification	Hiatt classification
RHA branch SMA	27	5.63	Type III	Type III
LHA branch LGA	13	2.71	Type II	Type II
Hepatomesenteric trunk	5	1.04	----------	--------
RHA branch SMA+LHA branch LGA	4	0.83	Type IV	Type IV
LHA accessory branch LGA	3	0.62	TypeV	Type II
RHA accessory branch SMA	2	0.4	Type VI	TypeIII
LHA aorta branch	1	0.2	----------	----------
LHA aorta branch + RHA branch SMA	1	0.2	----------	----------
LHA and RHA branch SMA	1	0.2	----------	----------
LHA branch CHA, absence of RHA	1	0.2	----------	----------
CHA branch of LGA	1	0.2	----------	----------
LHA aorta branch to branch in LGA and SMA	1	0.2	----------	----------
RHA bifurcated, absence of LHA	1	0.2	----------	----------
Confluence of PHA with RGA	1	0.2	----------	----------
Three hepatic arteries from aorta	1	0.2	----------	----------

RHA=right hepatic artery; LHA=left hepatic artery; SMA=superior mesenteric artery; LGA=left gastric artery; RGA=right gastric artery; CHA=common hepatic artery; PHA=proper hepatic artery

According to Michel´s classification, the most frequently observed abnormalities were: right hepatic artery of the superior mesenteric artery (Type III, n=27, 5.63%); left hepatic artery branch of the left gastric artery (Type II, n=13, 2.71%); and right hepatic artery arising from the superior mesenteric artery associated with the left hepatic artery arising from the left gastric artery (Type IV, n=4, 0.83%). Similarly, regarding the Hiatt classification, the most prevalent changes were: right liver accessory or substitute of the superior mesenteric artery (Type III, n=28, 6.05%), followed by accessory left hepatic artery or substitute of left gastric artery (Type II, n=16, 3.34%).

Fourteen patients (2.92%) had anatomical changes without defined classification. Among these, the most frequent was the hepatomesenteric trunk, present in five donors (1.04%). The other can be seen in Table 2, each being identified in only one patient (0.2%). 

## DISCUSSION

Knowledge of hepatic vascular anatomy is of great importance for the surgeon to perform abdominal intervention^6^. It is known that changes present at different stages of embryonic development lead to large variations in vasculature. In liver transplantation, in particular, detailed knowledge of the graft anatomy is essential to achieve its full arterialization and must be precisely identified at the time of organ captation^1^ ^,^ ^4^ ^,^ ^14^.

Thus, the classic vascular anatomy will serve as a guide to understanding the vascular supply and graft drainage^14^. In cases of anatomical variations, the hepatic lobes can receive blood supply from other vessels, as accessories, occurring in addition to the normal blood supply, or as a substitutive way, representing the only primary lobe arterial supply^3^.

Multiple anatomical variants were classified into 10 categories by Michels^8^ in 1966, in a study of 200 dissections, which is a reference to the present day for most studies^4^ ^,^ ^6^. This classification was modified by Hiatt in 1994^5^, which, unlike Michels^8^, did not make distinction between ancillary or hepatic arterial substitute structures, organizing it into six categories. Hiatt classification^5^ is simpler and frequently applied when the analysis is performed using angiographic studies, since it is considered difficult to distinguish between angiographically ancillary substitute or vascular structures^12^. In this study, both classifications were used.

According to the literature, the prevalence of anatomical variations ranges from 20-50%^4^. Zagyapan et al.^14^ analyzed 152 liver transplantation donors through digital angiography, finding 37,5% of anatomic variations of the hepatic artery^14^. Hiatt et al.^5^ in a series of 1000 patients who underwent liver transplantation, found 24.3% of hepatic changes^8^. In a series of 1200 cases, Kobayashi et al.^6^ identified normal hepatic arterial anatomy in 77.2%, and 22.8% of anatomic variations^6^. In this study, 416 patients (86.8%) had normal anatomy (Type I) and 63 patients (13.15%) some sort of variation, being this percentage the lowest prevalence among studies. 

According to Michels classification, the most frequent change according to the literature is the type III present in from 6-15.5% of cases^12^. It stands out as the most important because it has the potential to affect surgical procedures being indispensable its identification^4^. In agreement with the literature, this variation was also the most frequent in this sample, present in 5.63% of cases. The second most common is type II, reported in literature between 2.5-10% and observed in the present study in 2.71%. Type IV is described with an incidence of 1-7.4%^2^, and here it was found in 0.83%. The types VII, VIII, IX and X are rarely described in literature^12^ not being observed in this research. Regarding Hiatt classification, type III was seen in 6.05%, and type II in 16 cases (% 3:34), being them also the most prevalent in other studies^3^ ^,^ ^14^.

However, as anatomical variations may occur due to genetic aberrations in the embryonic period, no detailed classification can cover all types^6^. Rare anomalies not covered in Michels and Hiatt classifications were observed in 14 patients (2.92%), following a pattern of major studies. Koops et al.^7^ in their series revealed frequency of 1.8% rare presentations and non-classified; meanwhile, Ugurel et al.^12^ showed frequency of 3% of these forms in their paper. It is believed that the existence of rare variants shows that the embryological development of the branches of the aorta can be influenced by many factors, and is a complex process^12^.

Among these variations, the hepatomesenteric trunk, common hepatic artery and superior mesenteric artery originated from the aorta into a common trunk, and the common hepatic artery from the left gastric artery, are variants found rarely between abdominal vascular anomalies and also rarely reported in the literature. Chen et al.^2^ reported these variations in their series with a prevalence of 1.5-0.7%, respectively. In this series, these changes were found in 1.04 to 0.2%.

The precise knowledge of the most common and rare variations that produce different technical difficulties, or challenges, is essential to surgeons in order to avoid damage and vascular surgical complications^2^ ^,^ ^12^. Studies of hepatic arterial anatomy using case series of liver transplants show great diversity in the grafts, warning need for caution in surgical dissections, aiming effective arterialization and, consequently, success of the procedure in the receptor^1^ ^,^ ^3^ ^,^ ^9^ ^,^ ^11^ ^,^ ^13^.

## CONCLUSION

Detailed knowledge of hepatic arterial anatomy variations is of great importance to surgeons who perform approaches in this area, particularly in liver transplantation, since their identification and proper management are critical to the success of the procedure.
